# REFLECTIONS OF ADULTS TRANSITIONING TO LONG-TERM CARE FACILITIES: LOOKING BACK AND LOOKING FORWARD

**DOI:** 10.1093/geroni/igac059.2573

**Published:** 2022-12-20

**Authors:** Pei-Fen Chang, Gayle Hersch

**Affiliations:** Texas Woman's University, Houston, Texas, United States; Texas Woman's University, Houston, Texas, United States

## Abstract

Making a transition to a long-term care facility (LTCF) is often difficult for older adults. This study's intent was to explore ways in which life experiences and culture of older persons facilitate a successful transition to a LTCF. Eighteen participants were interviewed using a semi-structured interview guide to understand significant life events and personal factors impacting their lives. Interview data were analyzed using NVivo software to organize and manage emerging themes. The analysis revealed three related themes: person, current environments, and lifelong occupations. Within each major theme, subthemes were identified providing greater detail of how adaptive strategies are utilized to make such transitions positive. Participants were enabled to reflect upon their strengths and strategies available to adapt to these new residential settings. These perspectives are beneficial for healthcare practitioners to understand so as to personalize care and individualize the person's adjustment to a 'new' home.

